# Clear decisions: improved perceptual clarity reduces age-related decision-making deficits

**DOI:** 10.1093/geronb/gbag082

**Published:** 2026-05-10

**Authors:** Christopher Atkin, Hareth Al-Janabi, Stephen P Badham, Samuel J Perry, Katherine L Roberts

**Affiliations:** NTU Psychology, Nottingham Trent University, Nottingham, United Kingdom; Health Economics Unit, School of Health Sciences, College of Medicine and Health, University of Birmingham, Birmingham, United Kingdom; NTU Psychology, Nottingham Trent University, Nottingham, United Kingdom; Health Economics Unit, School of Health Sciences, College of Medicine and Health, University of Birmingham, Birmingham, United Kingdom; NTU Psychology, Nottingham Trent University, Nottingham, United Kingdom; (Psychological Sciences Section)

**Keywords:** Decision making, Applied Cognition, Perception, Vision, Cognition

## Abstract

**Objectives:**

Making effective everyday decisions is vital for maintaining well-being and independence in older age, yet older adults often face challenges in decision making due to age-related changes in perceptual and cognitive processes. Age-related declines in perception and cognition are not independent, in part because decoding impoverished perceptual input places additional demands on older adults’ limited cognitive resources. In this study, we test whether age-related deficits in decision-making performance can be reduced by improving the perceptual clarity of decision-making materials.

**Methods:**

In a pre-registered experimental study, 48 young (18–30 years) and 48 older adults (70+ years) completed decision-making, reading, and information-search tasks when written information was of standard clarity (moderate size and contrast) or enhanced clarity (increased size and contrast).

**Results:**

Older adults had worse decision-making performance than young adults, but, in line with the perceptual degradation hypothesis, the age deficit was reduced when decision-making information was clearer. Improved perceptual clarity also improved reading speed, but changes to reading speed did not fully account for the effect of clarity on decision-making performance.

**Discussion:**

The results suggest that improving perceptual clarity can reduce age-related deficits in decision-making through the provision of clear and accessible information with larger text and better contrast. Older adults can also take steps to improve their own perception, such as wearing appropriate glasses, using magnifiers, and reducing background noise.

As we age, decision-making tends to become slower, more effortful, and less efficient (Li et al., 2015), a change often attributed to declines in high-level cognition (e.g., working memory and attention), though the underlying mechanisms are likely multifaceted (Strough & Bruine de Bruin, 2020). Given that effective decision-making in later life is essential for managing personal healthcare, finances, and daily living, it is important to consider how older adults can be best supported in their decision-making. This is especially important in the context of an aging population, where maintaining autonomy and independence is a central goal for individuals, as well as a key objective of public health and social policy (WHO, 2024). If unaddressed, the gradual withdrawal from effortful cognitive engagement may reduce opportunities for older adults to maintain functional independence in everyday life.

Some forms of decision-making appear to be relatively preserved or even enhanced in older adulthood, likely due to crystallized abilities such as accumulated life experience and age-related emotional development (Del Missier et al., 2020). However, there are some everyday tasks where older adults consistently show age-related deficits, such as those that require applying complex decision rules to multi-attribute choices, which place greater demands on working memory and processing speed. One such task is the “Applying decision rules” (ADR) task from the adult decision-making competence test (A-DMC; Bruine de Bruin et al., 2007). In the ADR task, participants are asked to select a DVD player based on tables of information and a set of requirements (e.g., Brian selects the DVD with the highest number of ratings greater than “Medium”). Typically, older adults show worse performance on this task than young adults (Bruine de Bruin et al., 2015; Del Missier et al., 2013). Performance on the ADR task is linked to working memory and numeracy ability (Del Missier et al., 2013), both of which decline in older age (Bruine de Bruin et al., 2015), and is likely to also be affected by older adults’ tendency to seek less information, rely more on gist, choose options that are “good enough,” and avoid cognitively demanding tasks (e.g., Nolte & Löckenhoff, 2023). The task also relies on visual perception to read task scenarios and extract information from printed tables.

Aging is associated with declines in numerous aspects of vision and hearing, including reduced visual acuity and visual contrast sensitivity (Greene & Madden, 1987) and elevated hearing thresholds and impaired auditory temporal processing (Humes et al., 2009). In older adults, impaired vision and hearing are associated with worse cognition, a faster rate of cognitive decline, and an increased risk of dementia (Roberts & Allen, 2016; Uchida et al., 2019). While the exact mechanisms underlying these links are yet to be fully understood, there is clear evidence that decoding impoverished perceptual input is cognitively demanding (Roberts & Allen, 2016), reducing capacity to perform other cognitive tasks (Monge & Madden, 2016; Pichora-Fuller & Singh, 2006). Typically, experimental studies of decision-making present information that is clearly visible and/or audible to participants. However, these optimal conditions may mask the extent to which older adults struggle with decision-making in real-world scenarios, where information needed for decisions can be small, degraded, or presented in a noisy environment (for example, nutrition labels on food packaging, bus timetables, or unclear phone lines).

According to the *information degradation hypothesis* (Monge & Madden, 2016; Schneider & Pichora-Fuller, 2000), improving perceptual clarity should reduce demands on perceptual-cognitive processes, thus freeing up resources for a cognitive task. Support for the hypothesis comes from studies showing that degrading the perceptual input has a negative impact on a number of cognitive processes (e.g., Atkin et al., 2024; Boutet & Meinhardt-Injac, 2019; Laudate et al., 2012; Toner et al., 2012), including those directly linked to decision-making, such as working memory (Billig et al., 2020) and processing speed (digit cancellation; Toner et al., 2012). Visual degradation also slows reading speed and reduces comprehension (Mitzner & Rogers, 2006; Warrington et al., 2018), which is relevant when decisions are based on written information.

The information degradation hypothesis further predicts that older adults will be more affected by changes in perceptual clarity than young adults due to age-related perceptual decline and reduced capacity to compensate for loss of clarity. Age-related declines in perception and perceptual processing speed can amplify the impact of poor clarity, while deficits in top-down cognitive control and working memory limit older adults’ ability to overcome the lack of clarity. Support for this part of the hypothesis appears to be specific to the cognitive process and task design/difficulty. Some studies do find this predicted interaction between age and perceptual clarity (Allen et al., 2017; Boutet & Meinhardt-Injac, 2019; Cronin-Golomb et al., 2007; Laurienti et al., 2006). Critically, Cronin-Golomb et al. (2007) found that older adults were more affected by clarity than young adults on letter identification and word reading tasks. However, other studies have found that young and older adults are equally affected by changes in perceptual clarity (Atkin et al., 2024; Billig et al., 2020; Laudate et al., 2012; Toner et al., 2012). To our knowledge, no studies have directly assessed the impact of perceptual clarity on decision-making in young and older adults.

If poor perceptual clarity negatively affects the speed, effortfulness, and accuracy of older adults’ decisions, it would indicate a need for environmental support during decision-making tasks (Craik, 2022). In healthcare, guidance on the development of Decision Aids (Decision Support Tools) emphasizes the use of clear perceptual information to convey risks, as well as taking into account any communication difficulties (Stacey et al., 2024). Decision Aids are typically found to improve patients’ satisfaction with their decisions and decrease decisional conflict (e.g., Stacey et al., 2024). Similar benefits for everyday decisions, such as choosing nutritional food or planning a journey, could be achieved through the provision of clearer decision-making materials or through older adults taking steps to improve their own perception by wearing appropriate glasses or hearing aids, increasing the size of stimuli through magnifiers or adjusting screen size, or reducing background noise. These steps could prevent a downward spiral where worsening perception and cognition lead to detrimental lifestyle changes for older adults, which in turn exacerbate perceptual and cognitive decline.

Environmental support refers to external cues and structures that reduce the cognitive demands of tasks, especially when self-initiated processing is limited (Craik, 2022). This concept has traditionally been applied to memory, where contextual prompts and cues significantly enhance recall, particularly in older adults (e.g., Badham et al., 2023). However, decision-making is equally vital for maintaining well-being and independence in later life (Usher & Stapleton, 2022). Therefore, there is an increasing need to extend the principles of environmental support to decision-making contexts. This involves designing perceptually clean environments that reduce cognitive load so that individuals are better able to make accurate decisions, even when their internal cognitive resources are compromised.

The aim of the present study was to establish whether improving the perceptual clarity of written information alleviates age-related deficits in everyday decision-making. Two decision-making tasks were employed. The first was a purchasing decision task adapted from the “applying decision rules” subtest of the A-DMC (Bruine de Bruin et al., 2007). The second was a nutrition decision task adapted from the U.K. version of the Newest Vital Sign health literacy test (Rowlands et al., 2013). Both are established measures used to assess the speed and accuracy of responses in young and older participants and, to some extent, represent the types of everyday decisions made by older adults. Both tasks were presented with “standard” clarity (moderate size and contrast) and in an “enhanced” format (larger size, higher contrast).

In line with the information degradation hypothesis, we predicted that older adults would benefit more from the perceptually enhanced scenarios than young adults. We additionally measured the impact of perceptual clarity on self-paced reading speed and information search to assess whether clarity affects decision-making performance over and above any changes in reading and searching for information.

We assessed both speed and accuracy of decision-making (see Supplementary Material) and primarily report Balanced Integration Scores (BIS), which combine both. The combined response-time (RT) and accuracy measure accounts for speed-accuracy trade-offs, which are known to differ in young and older adults (Salthouse, 1979). Older adults are sometimes able to compensate for age deficits through increased effort and/or alternative strategies, allowing them to achieve comparable accuracy to young adults (Craik, 2022). To some extent, a pure accuracy measure would reflect older adults’ real-life experience of decision-making, in which few tasks are time-limited. However, this does not take into account the broader consequences of slower, more effortful decision-making, such as older adults’ tendency to avoid, delay, and defer decisions as they become more cognitively demanding (Crawford et al., 2023; Nolte & Löckenhoff, 2023).

We additionally assessed a number of factors known to affect decision-making ability to evaluate whether they (a) predict performance on the decision-making tasks and (b) are associated with the amount of benefit participants receive from improved perceptual clarity. Steps to improve perceptual clarity could prove particularly useful for groups known to have worse decision-making ability, including those with lower socioeconomic status (SES; Bruine de Bruin et al., 2007), lower educational attainment (Boyle et al., 2013), and reduced cognitive abilities linked to decision-making, including numeracy (Skagerlund et al., 2022), processing speed (Del Missier et al., 2017), and possibly vocabulary (Lindow & Lang, 2022).

## Method

### Transparency and openness

We report how we determined our sample size, data exclusions, manipulations, and measures in the paper. Pre-registration (https://osf.io/dw3au/overview?view_only=e6c7a6be41c24532b599257f8cd41416), data, analysis code, and research materials (https://osf.io/myb3g) are available at the Open Science Framework.

### Design

The study employed a 2 × 2 mixed design, with a between-participants factor of age group (young, older adults) and a within-participants factor of clarity (standard, enhanced). The experimental tasks assessed purchasing decisions, nutrition-based decisions, reading, and information search under standard (moderate size and contrast) and enhanced (larger size, higher contrast) conditions. Additional measures are reported in Table 1.

**Table 1:** Participant demographics and descriptive statistics, with results of *t*-tests.

Variables	Young		**Older**		Group difference		
	*M* (*SD*)	*N*	*M* (*SD*)	*N*	*t (n)*	*p*	*d*
**Age**	25.48 (3.29)	48	76.85 (3.46)	48			
**Gender**							
Female		25		24			
Male		23		24			
**Education**	6.15 (1.85)	48	5.77 (2.11)	48	−0.93 (94)	.356	−0.19
**Socioeconomic status**	4.52 (3.02)	33	7.16 (2.13)	43	4.46 (74)	<.001	1.03
**Processing speed**	14.98 (3.58)	48	9.96 (3.59)	47	−7.48 (93)	<.001	−1.53
**Vocabulary**	0.51 (0.15)	48	0.65 (0.10)	48	5.38 (94)	<.001	1.10
**Numeracy**	0.62 (0.24)	48	0.49 (0.20)	48	−2.83 (94)	<.001	−0.58
**Vision**	3.73 (0.87)	48	3.60 (0.77)	48	−0.75 (94)	.456	−0.15
**Hearing**	4.19 (0.76)	48	3.88 (1.00)	48	−1.79 (94)	.089	−0.31

*Note*. Except for age and gender, higher scores on all measures are better. Self-reported vision and hearing were rated on a five-point scale.

### Participants

Forty-eight young (18–30) and 48 older (70+) participants were recruited via Prolific (prolific.com). An additional six (two older and four younger) participants were excluded from analysis for failing attentional checks and/or for overly erroneous responses. Participants completed the study online and received £9.00 compensation. Sample sizes were determined using a power analysis based on previous research, as outlined in the preregistration. Table 1 shows participant demographics and additional cognitive measures. All reported normal or corrected-to-normal vision. Participants were native English speakers. They were required to be resident in England to enable the use of the English Index of Multiple Deprivation (IMD) to assess SES. The exclusion criteria were self-reported memory impairments/impaired cognitive function, learning difficulties, and color blindness. Cognitive function was assessed via the Letter Comparison task (processing speed; Salthouse & Babcock, 1991), the Mill Hill Vocabulary Test (crystallized intelligence; Raven et al., 1988), and a numeracy test (objective numeracy; Weller et al., 2013). The study was given a favorable opinion by Nottingham Trent University’s Business, Law and Social Sciences Research Ethics Committee.

### Materials

All tasks were built using Gorilla Experiment Builder (Anwyl-Irvine et al., 2020) and completed on the participant’s own computer. To account for differences in screen size, participants completed a calibration step in which they matched an on-screen rectangle to a standard-size credit card. Participants entered their viewing distance if different from 50 cm. Stimuli were presented in Helvetica font at 0.47° (∼12.31 px; ∼12.2 pt font size) in the standard condition and 0.57° (∼14.93 px; ∼14.8 pt font size) in the enhanced condition. All tasks were presented on a white background (RGB: 255, 255, 255). Standard stimuli were presented in light gray (RGB: 219, 219, 219), while enhanced stimuli appeared in black (RGB: 0, 0, 0), based on prior research investigating reduced text contrast in young and older adults (Warrington et al., 2018). Piloting ensured standard stimuli were suprathreshold, and all participants passed a text-entry screening task to confirm legibility.

#### ADR task

A purchasing decision task was adapted from the “Applying Decision Rules” (ADR) task from the A-DMC (Bruine de Bruin et al., 2007). Participants were presented with tables of data to guide decisions about purchasing a TV (e.g., ratings of picture quality and sound quality) and a laptop (e.g., ratings of screen resolution and battery life). For each scenario (TV, laptop), participants were presented with 10 questions (5 standard clarity and 5 enhanced) in which they were asked to select the product(s) that met a hypothetical set of preferences (e.g., the best picture quality). The TV questions were taken directly from the ADR task (e.g., Brian selects the TV with the highest number of ratings greater than “Medium”). For the laptop task, the hypothetical person’s name was changed, and categories were adjusted to match laptops (e.g., battery life replaced sound quality). Tables contained the same information in both tasks, but for the laptop task, the columns were shifted down and to the left so that values appeared in different positions. Scores were calculated for each clarity condition as the proportion correct (PC) out of 10 (5 TV and 5 laptop questions) and the median response time on correct trials.

#### Nutrition decision task

The Nutrition Decision task was adapted from the U.K. version of the Newest Vital Sign health literacy test (Rowlands et al., 2013). In this task, participants are presented with a nutrition label to guide decisions about ice cream and granola bars (e.g., the amount of fat and sugar per portion). For each scenario, two of the questions required an evaluative decision (e.g., “Is it safe for [a person with a peanut allergy] to eat this ice cream?”; “A food is considered unhealthy if it contains more than 8 g of fat or 10 g of sugar per serving. Would this food be considered unhealthy?”). The other four required a numerical calculation that could feed into a nutritional choice (e.g., “If you are advised to eat no more than 60 grams of carbohydrate for dessert, what is the maximum amount of ice cream you could have?”; “If you are advised to limit your sugar intake to 30 grams per day, how many granola bars can you eat while staying within your limit?”). The Ice Cream questions were taken directly from Questions 1 to 5 from the Newest Vital Sign health literacy test (Rowlands et al., 2013), plus one additional question (“A food is considered healthy if it contains less than 0.1 g of sodium and less than 1.5 g of saturated fat per serving. Would this ice cream be considered healthy?”). The granola bar questions were similar to the ice cream questions (https://osf.io/myb3g). Scores were calculated for each clarity condition as the PC and median response time on correct trials.

#### Reading task

The self-paced reading task followed Mitzner and Rogers (2006). Participants read a total of 20 sentences (10 standard clarity and 10 enhanced), presented one word at a time. Each sentence was preceded by a fixation cross. Participants initiated each trial by pressing the space bar, which triggered the word-by-word display of a sentence. Words appeared sequentially with each additional space bar press, encouraging participants to read at their own natural pace.

The stimuli consisted of a set of simple, syntactically correct English sentences of between 7 and 9 words, e.g., “Jill looked back through the open window.” Six (three standard clarity and three enhanced) sentences were followed by a yes/no comprehension question to ensure attention and understanding (e.g., “Did Jill look back through the open window?”). Median response times per word were calculated for each sentence, separately for each participant and condition.

#### Information-search task

Participants were presented with tables of information similar to those in the ADR task but with different numerical values. On each trial, they were instructed to count the total number of occurrences of a specific target number (e.g., “find all the 5s”) within the table and enter the total in a text box. Participants completed eight trials in random order (four standard clarity and four enhanced). Median search time was calculated for each condition.

#### Additional measures

Participants additionally completed online versions of the Mill Hill Vocabulary test (Raven et al., 1988; score out of 33), numeracy test (Skagerlund et al., 2022; score out of 8), and a Letter Comparison Task (Salthouse & Babcock, 1991; score out of 60).

### Procedure

All participants received an information sheet and consent form and were asked to create a unique identifier and provide demographic details (see Table 1).

Participants then completed a fixed sequence of tasks. The fixed order ensured a clear gap between the first and second sets of decision-making questions and mirrored the fixed order of tasks in the Adult Decision Making Competence test (Bruine de Bruin et al., 2007). The first ADR task involved either a TV or laptop scenario, with scenario type counterbalanced across participants. This was followed by the first Nutrition task, in which participants completed either the Ice Cream or Granola Bar version, similarly counterbalanced. Next, participants completed the Information-search task, Reading task, Letter Comparison task, Numeracy task, and Vocabulary task. The experiment concluded with the second ADR task (featuring the scenario not completed in the first instance) and the second Nutrition task (again featuring the scenario not completed in the first instance). Within the ADR and Nutrition tasks, questions alternated between standard and enhanced presentation, with initial clarity counterbalanced across participants. On all tasks, participants were instructed to respond as quickly and as accurately as possible. The experiment took approximately 60 min to complete.

## Results

### Data preparation and statistical analysis

Analyses were completed in IBM’s SPSS Statistics version 29, and data organization and cleaning were completed using RStudio 2024.09.0. Data and analysis code are openly available (https://osf.io/myb3g).

The main dependent variable for the decision-making tasks was the BIS (Liesefeld & Janczyk, 2019). Balanced Integration Score standardizes accuracy and reaction time, integrating them into a single efficiency measure that accounts for speed-accuracy trade-offs (Liesefeld & Janczyk, 2019), which are well-established and known to vary with age, with older adults prioritizing accuracy over speed (e.g., Rabbitt, 1979). Balanced Integration Score is derived by calculating *z* scores for PC and median response times on correct trials ($\tilde{RT}$), across all participants (i) and conditions (j), and then subtracting the RT *z* score from the proportion-correct *z* score. Higher BIS values indicate better performance.


$${\mathrm{BIS}}_{i,j}=\;z_{i,j}^{PC}-\;z_{i,j}^{\tilde{RT}}=\;\frac{{PC}_{i,j}-\bar{PC}}{{SD}^{PC}}-\;\frac{{\tilde{RT}}_{i,j}-\bar{\tilde{PC}}}{{SD}^{\tilde{RT}}}$$

For reaction-time analyses, younger adults’ reaction times were adjusted following the linear transformation method proposed by Madden et al. (1992) to account for age-related slowing (see Supplementary Material for more information).

Data from the decision-making (BIS), reading (transformed RT), and information-search (transformed RT) tasks were analyzed using 2 × 2 mixed ANOVAs, contrasting age group (between-participants: young, older) and clarity (within-participants: standard, enhanced). Effect sizes for ANOVAs are reported using partial eta squared (η_p_^2^). Significant interactions were assessed using post hoc analyses with Bonferroni corrections for multiple comparisons, with effect sizes reported using Cohen’s *d*.

**Figure 1:**
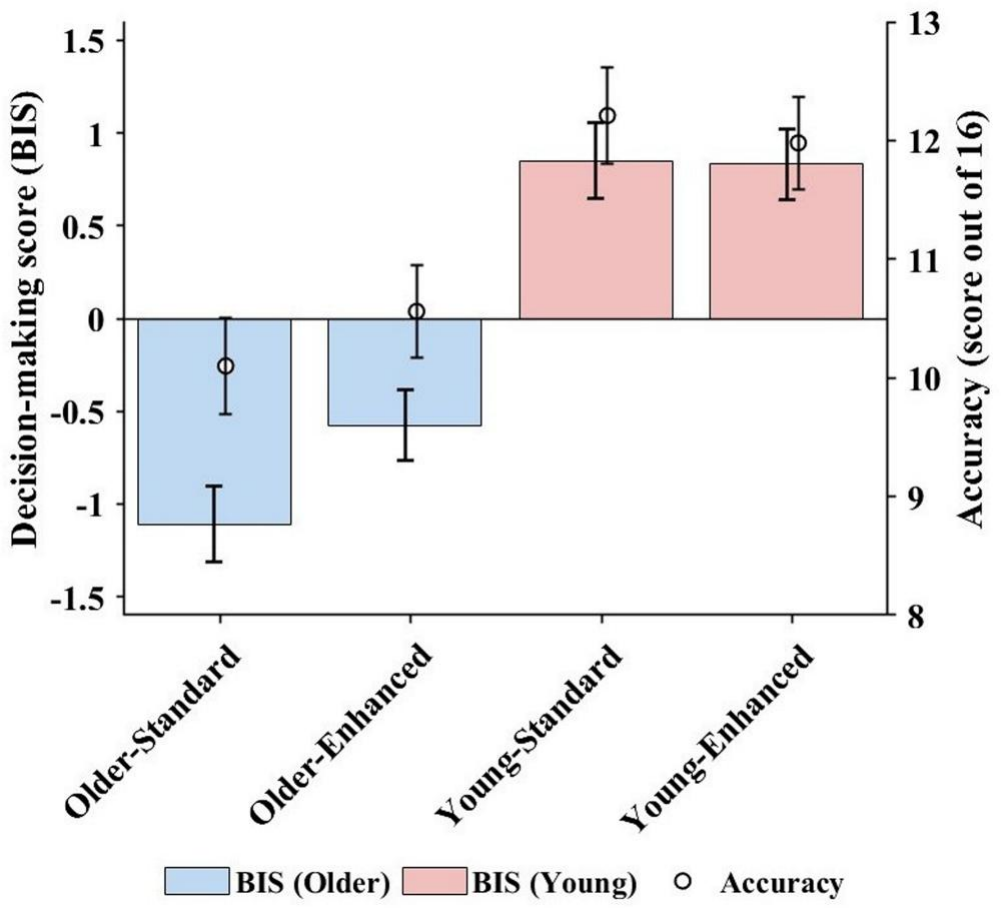
Decision-making performance (BIS) as a function of age group (older, young) and perceptual clarity (standard, enhanced). BIS = Balanced Integration Score. Higher BIS equals better performance. Accuracy is overlaid for reference. Error bars indicate standard errors.

A 2 (age group: young, older adults) × 2 (clarity: standard, enhanced) mixed ANOVA was conducted on BIS across both decision-making tasks (see Figure 1 for mean BIS across conditions). Young adults had better decision-making performance than older adults, *F*(1, 94) = 40.66, *p* < .001, η_p_^2^ = 0.30, and decision-making was better with enhanced clarity than standard clarity, *F*(1, 94) = 7.42, *p* = .008, η_p_^2^ = 0.07. There was a significant interaction, *F* (1, 94) = 8.44, *p* = .005, η_p_^2^ = 0.08, which showed that clearer information improved decision-making for older adults (*p* < .001, *d* = 0.47), but not young adults (*p* = .898, *d* = 0.03). The same general pattern was seen in separate analyses of response times and accuracy, and in separate analyses for the ADR and nutrition tasks (see Supplementary Material).

### Reading task

**Figure 2:**
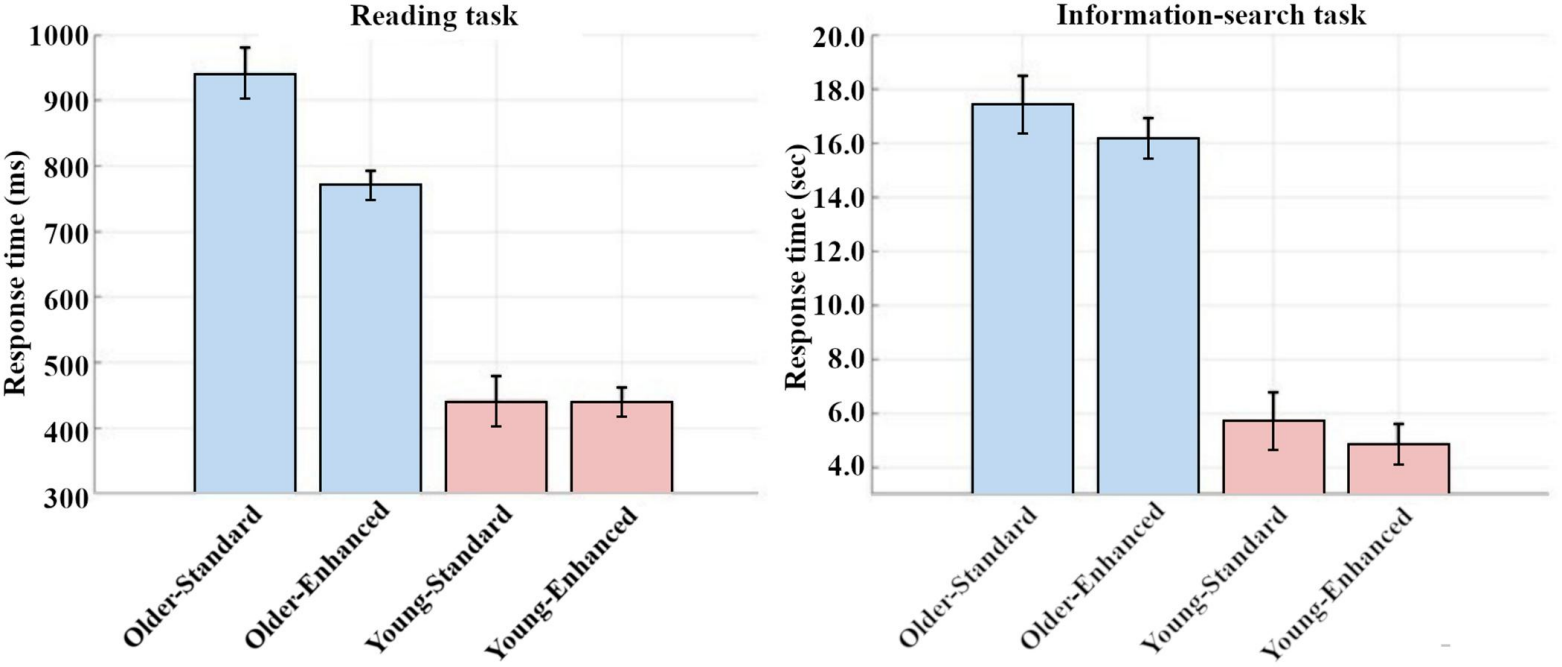
Transformed response times for the reading task (milliseconds per word) and information search task (seconds per table), as a function of age group (older, young) and perceptual clarity (standard, enhanced). Error bars indicate standard errors.

A 2 (age group: young, older) × 2 (clarity: standard, enhanced) mixed ANOVA was conducted on the transformed response time (Madden et al., 1992) for self-paced reading speed (see Figure 2 for means across conditions). One participant’s median response time was more than 5 *SD*s above the group mean and was excluded from the analysis (note: the pattern of findings remained unchanged with the participant included in the analysis). Older adults had slower reading times than young adults, *F*(1, 93) = 117.32, *p* < .001, η_p_^2^= 0.56, and reading times were slower for standard sentences than enhanced sentences, *F*(1, 93) = 14.53, *p* < .001, η_p_^2^ = 0.14. There was a significant interaction, *F*(1, 93) = 14.07, *p* < .001, η_p_^2^ = 0.13, which showed that reading speed was faster for enhanced sentences than standard sentences for older adults (*p* < .001, *d* = 0.55) but not young adults (*p* = .965, *d* = 0.023). Responses to the attention-check questions were highly accurate, with only four participants responding incorrectly to one of the six questions.

### Information-search task

A 2 (age group: young, older) × 2 (clarity: standard, enhanced) mixed ANOVA was conducted on the transformed response time (Madden et al., 1992) for information search speed (see Figure 2 for means in each condition). Young adults responded more quickly than older adults, *F*(1, 94) = 89.47, *p* < .001, η_p_^2^= 0.49, and participants responded more quickly when information was enhanced than when it was standard clarity, *F*(1, 94) = 4.69, *p* = .033, η_p_^2^ = 0.05. There was no significant interaction, *F *< 1, *p* = .676, η_p_^2^ = 0.002. Information search was highly accurate: 39 young and 38 older adults made no mistakes on the eight trials. Seven older adults and nine young adults made one mistake, and three older adults made two mistakes. Mistakes were distributed across both clear (*n* = 11) and unclear (*n* = 9) conditions.

### Mediation analysis

An exploratory within-participants mediation analysis was conducted using MEMORE v2.1 (Montoya & Hayes, 2017) to test whether reading speed (*z* score) and information-search speed (*z* score) mediated the effect of clarity (standard vs enhanced) on decision-making performance (BIS) for older adults only. Bootstrapping with 5,000 resamples was used to generate bias-corrected 95% confidence intervals for the indirect effects.

**Figure 3:**
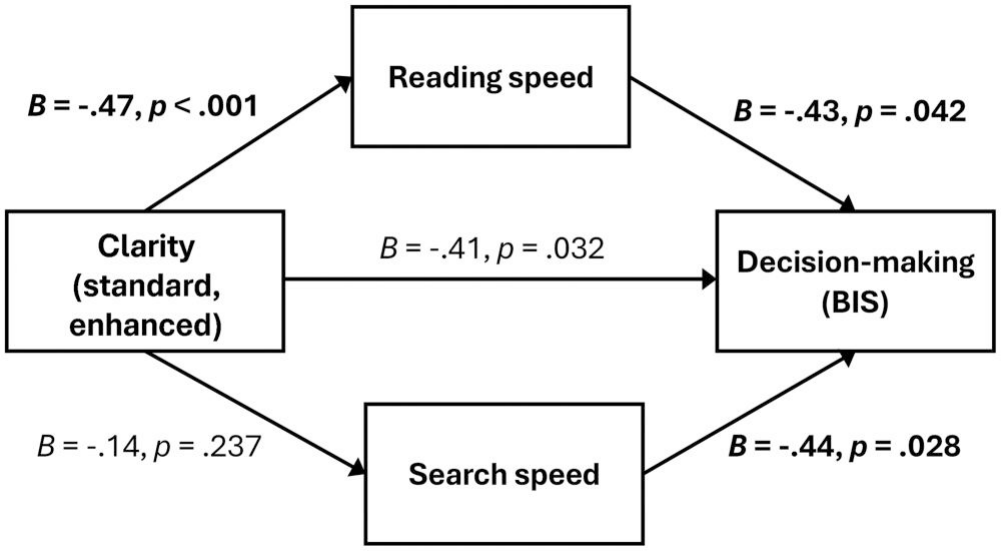
A within-participant mediation model: the effect of perceptual clarity on BIS decision-making performance and the mediation effect of reading speed and search speed in older adults. BIS = Balanced Integration Score.

The total effect of clarity on decision-making performance was significant, *B* = –0.55, *t*(46) = –3.27, *p* = .002. When both mediators were included in the model, the direct effect remained significant, *B* = –0.41, *t*(44) = –2.22, *p* = .032. The indirect effect via reading speed was significant, *B* = –0.20, 95% CI [–0.40, –0.02], while the indirect effect via information-search speed was not significant, *B *= 0.06, 95% CI [–0.06, 0.17]. These results indicate that the effect of clarity on decision-making performance is partially mediated by reading speed, but not by information-search speed. Figure 3 shows the mediation model with statistics for each path.

### What predicts decision-making ability and the effect of clarity?

Exploratory multiple linear regressions were conducted to assess whether age group, education, SES, numeracy, vocabulary, and processing speed predicted decision-making performance (average BIS across standard and enhanced clarity conditions) and the amount of benefit participants received from improved perceptual clarity (difference between BIS in the standard and enhanced conditions). In both regression models, the Variance Inflation Factors were less than 2, suggesting no need to correct for multicollinearity.

The overall model predicting BIS was significant, *F*(6, 74) = 14.74, *p* < .001, and explained 55.8% of the variance in average BIS (*R*^2^ = 0.558, adjusted *R*^2^ = .519). Better average BIS were associated with being in the younger age group (*B *= 0.930, β = 0.298, *p* = .010), higher numeracy scores (*B *= 3.041, β = 0.461, *p* < .001), higher vocabulary scores (*B *= 2.094, β = 0.192, *p* = .042), and faster processing speed (*B *= 0.106, β = 0.280, *p* = .010). BIS was not significantly predicted by educational attainment (*B *= 0.028, β = 0.037, *p* = .670) or SES as indexed by deprivation decile (*B *= 0.040, β = 0.074, *p* = .435). There were no significant predictors of the amount of improvement in BIS with clearer perceptual information, and the overall model was not significant (*F*(6, 74) = 1.82, *p* = .107). This was also true when analyses were conducted separately for the young and older age groups (see Supplementary Material).

## Discussion

The present study investigated whether improving the clarity of decision-making materials alleviates age deficits in everyday decision-making. Young and older adults completed decision-making tasks where the questions and tables of information were of “standard” clarity (moderate contrast and size) or “enhanced” clarity (higher contrast, larger size). As expected, young adults had better decision-making performance than older adults. While young adults’ decisions were unaffected by perceptual clarity, older adults performed better when information was clearer, with a moderate effect size (*d* = 0.47). Improved perceptual clarity, therefore, reduced the age-related deficit in decision-making. The benefit of clear information was partially mediated by the effect of clarity on reading speed, but a direct link between clarity and decision-making remained. These findings underline the importance of ensuring that older adults are able to access clear decision-making information in everyday life.

Our findings show that perceptually clearer forms of information significantly enhance decision-making in older adults, demonstrating for the first time that improved perceptual clarity can reduce age deficits in decision-making. These findings support the information degradation hypothesis (Monge & Madden, 2016; Pichora-Fuller & Singh, 2006; Roberts & Allen, 2016), which suggests that when perceptual information is degraded, the additional processing demands and impoverished representations lead to impaired cognitive performance. Older adults are predicted to be more affected by perceptual clarity than young due to age-related slowing and reduced capacity to compensate for reductions in perceptual clarity. In contrast, young adults’ intact systems may enable them to maintain decision-making performance despite the reduced clarity, either through preserved perceptual processing (Humes et al., 2013) or more effective compensatory strategies (Radnan et al., 2023).

Some studies do find a greater effect of clarity for older adults, as predicted by the information degradation hypothesis, while others find that perceptual clarity affects young and older adults equally. Our findings echo this variability. Older adults benefited more than young adults from perceptual clarity on the decision-making and reading tasks, but not the information search task, in which both age groups were similarly affected by clarity. A common thread seems to be that young and older adults are similarly affected by clarity on tasks that involve feature-based attentional guidance: information search (here), visual search (Laudate et al., 2012), color discrimination (Atkin et al., 2024), and digit cancellation (Toner et al., 2012). This kind of expectation-driven search is known to support performance in older adults by reducing reliance on bottom-up input (Madden, 2007). Tasks requiring reading or more complex cognitive processes seem more likely to show an interaction between age and clarity: reading and decision-making (here), reading and picture naming (Cronin-Golomb et al., 2007), and face processing (Boutet & Meinhardt-Injac, 2019).

For older adults, the link between perceptual clarity and decision-making performance was partially mediated by the effect of clarity on reading speed. While this finding needs to be replicated in a larger sample of older adults, the significant indirect effect of reading speed provides support for concerns that cognitive impairment is overdiagnosed in older adults with sensory deficits (Abraham et al., 2023). The data here suggest that simply reducing the clarity of information necessary for efficient reading is sufficient to impair decision-making performance. This aligns with research showing that less-clear sensory input, such as that caused by reduced vision and hearing, can lower neuropsychological test scores and thus inflate diagnoses of cognitive decline (Dumassais et al., 2024). However, the residual direct effect of clarity on decision-making is consistent with the idea that perceptual degradation increases the cognitive load (Monge & Madden, 2016; Pichora-Fuller & Singh, 2006; Roberts & Allen, 2016) by consuming resources that would otherwise be available for higher-order processes (Billig et al., 2020). Future research should therefore ensure that perceptual ability is accounted for and that task-related perceptual clarity is optimal before interpreting cognitive test performance, particularly in aging populations where sensory decline is common (Vázquez-Sánchez et al., 2025).

Unexpectedly, neither decision-making performance nor the improvement in decision-making with improved perceptual clarity was associated with educational attainment or SES. This may be due to insufficient power for the exploratory regression analyses. In addition, this older-adult sample had higher educational attainment than the general population (33.8% had a Level 4+ qualification compared with 15%–20% of 70+ older adults in the 2021 UK Census). The elevated education level in this sample may mask costs linked to reduced formal education in the broader population. Socioeconomic status was assessed using the English IMD deciles. IMD deciles are based on small geographic areas of around 650 households (“Lower-layer super output areas”). While IMD deciles provide a useful alternative to job- and salary-based measures of SES for retired adults, they may not be sufficiently specific to individuals for smaller-scale studies.

The decision-making tasks used here incorporate features of small, everyday consumer and nutrition choices that older adults may regularly encounter, as opposed to major life decisions that might be supported by specific Decision Aids. The ADR (purchasing) task (Bruine de Bruin et al., 2007) is designed to assess decision strategies such as satisficing and elimination by aspects. Older adults show consistent deficits on this task relative to young adults, and importantly, performance on the ADR task is correlated with self-reported real-world decision-making success, as measured by the Decision Outcomes Inventory (Bruine de Bruin et al., 2007). However, the present study still represents a simplified decision environment. Real-world decisions often involve incomplete or uncertain information, multiple acceptable outcomes, and greater ambiguity than the current tasks allow. Moreover, in everyday life, people can choose to make a decision over a longer period of time and draw on everyday supports such as notes, lists, or reminders; simple technological tools such as calendars or smartphones; and discussions with friends and relatives (Perry et al., 2026). Given that the current study clearly demonstrates an impact of perceptual clarity on older adults’ decision-making, further research should evaluate the relative impact of clarity for diverse types of decisions and decision-making contexts. Moreover, given the prevalence of age-related hearing loss, it would be valuable to assess the impact of auditory clarity when decisions are based on spoken information.

As the study was conducted online, there was limited control over display settings such as monitor resolution and lighting. As a result of this, plus natural variation in (corrected) visual acuity and contrast sensitivity, the perceived visual clarity of the tables will have varied across participants. This variation was minimized through piloting and in-experiment checks that ensured the standard stimuli were suprathreshold for all participants and by utilizing within-participant manipulations of perceptual clarity. In addition, a scaling function was used to ensure that all visual information was presented at a consistent size across monitors (Anwyl-Irvine et al., 2020). Controlled lab-based studies, in combination with measures of age-related visual decline, could provide more accurate insights into the relative impact of poor clarity for older compared with young adults and whether the effect of clarity on cognitive processes is greater for those with initially poorer visual perception. However, as participants in the present study were using their usual monitor settings, room lighting, and glasses or contact lenses, the present findings provide an accurate estimate of the amount of impact that a small change in perceptual clarity can have on young and older adults’ everyday decision-making.

The current findings support Craik’s (2022) environmental support framework by showing that enhancing perceptual information improves older adults’ decision-making. This suggests that age-related cognitive deficits can be offset by structured external support. By providing clear sensory input, our results demonstrate that perceptual scaffolding can effectively compensate for age-related deficits in everyday tasks. The findings have clear implications for businesses, health providers, and public organizations. Presenting information in a perceptually clear format goes beyond accessibility and serves as a form of cognitive intervention for older adults. While many organizations (e.g., healthcare providers) strive to produce clear information, older adults still encounter a wide array of unclear information in everyday life, such as nutrition labels on food and public transport timetables. In these circumstances, older adults could benefit from taking steps to improve their own perceptual clarity through, for example, wearing correct glasses for the task, using magnifiers, and seeking better lighting. Provision of clear decision-making materials, combined with steps to improve perceptual clarity, can help reduce avoidable errors, improve decision outcomes, and empower older adults to retain independence in everyday contexts.

In conclusion, the present findings demonstrate that improved perceptual clarity can reduce the age-deficit in decision-making performance, consistent with the information degradation hypothesis. These results have practical implications for how older adults access decision-making materials and underline the importance of considering perceptual factors when designing research and interventions to address age-related cognitive decline.

## Supplementary Material

gbag082_Supplementary_Data

## Data Availability

The research was preregistered, and the preregistration is available at: https://osf.io/dw3au/overview?view_only=e6c7a6be41c24532b599257f8cd41416. Data, analysis code, and research materials are available at: https://osf.io/myb3g.
