# Supplementary material for: Clear decisions: improved perceptual clarity reduces age-related decision-making deficits
Source: J Gerontol B Psychol Sci Soc Sci. 2026 May 10;81(7):gbag082. doi: 10.1093/geronb/gbag082 (PMC13294560; doi:10.1093/geronb/gbag082)
Supplement: gbag082_Supplementary_Data [file gbag082_supplementary_data.pdf]

The Journals of Gerontology, Series B: Psychological Sciences and Social Sciences
Supplementary Material: Atkin et al. Clear decisions: improved perceptual clarity reduces age-related decision-making deficits.

Pre-registration link

OSF | The Effect of Clarity in Easing Everyday Decisions

Overview

The analysis is presented in accordance with the pre-registration. The analysis for each respective task is presented in the following order: Combined (ADR and nutrition tasks), ADR task, Nutrition task, Self-paced Reading task, and Information Search task. The combined data, The ADR task and Nutrition task data includes a 2 × 2 mixed ANOVA (Age Group [between-participants]: Young, Older adults × Clarity [within-participants]: Standard, Enhanced) on Balanced Interaction Score (BIS; Liesefeld & Janczyk, 2019), transformed response times (Madden, Pierce, & Allen, 1992), and then separately on accuracy. For the Self-paced Reading task and Information Search task results include a 2 × 2 mixed ANOVA (Age Group [between-participants]: Young, Older adults × Clarity [within-participants]: Standard, Enhanced) on transformed response times (Madden et al., 1992).

For reaction-time analyses, young adults' reaction times were adjusted following the linear transformation method proposed by Madden et al. (1992) to account for age-related slowing. In this approach, the relationship between young and older adults' mean RTs is modelled as a linear function, $RT_{old} = m(RT_{young}) + b$, where the slope m represents proportional slowing and the intercept b captures an additive constant. Transforming the young adults' RTs using this function aligns their overall response speed with that of the older adults while preserving relative differences across task conditions, allowing age-related effects on specific cognitive processes to be evaluated independently of generalised slowing.

Post hoc analyses using Bonferroni corrections were used to investigate significant interactions with effect sizes reported using Cohen's *d*. Effect sizes for ANOVAs are reported using partial eta squared. Bayes factors (BF_{10}) were calculated using JASP software (Love et al., 2015) to measure evidence for the null versus alternative hypotheses for ANOVAs and t tests. The Bayes factor provides an odds ratio for these hypotheses, where values less

than 1 favour the null hypothesis and values greater than 1 favour the alternative hypothesis. Previous research has categorized the strength of Bayes factors with labels such as "substantial" (3-10), "strong" (10-100), and "decisive" (100+) (Wetzels et al., 2011). The means and standard errors for BIS, transformed response time and accuracy can be seen in Table S1, and the ANOVAs and post hoc analysis can be found in Table S2.

Table S1. Means and standard deviations (in parentheses) for BIS, transformed response time (RT), and accuracy across task, age group (young vs. older), and clarity condition (standard vs. enhanced).

		**Older**		**Young**	
		**Standard**	**Enhanced**	**Standard**	**Enhanced**
**Combined**	BIS	-1.11 (1.52)	-.57 (1.40)	.85 (1.31)	.83 (1.26)
	RT	54313.07 (22904.18)	47229.50 (14793.22)	26466.26 (3796.73)	26094.61 (2925.45)
	Accuracy	10.10 (2.94)	10.56 (2.87)	12.21 (2.68)	11.98 (2.53)
**ADR**	BIS	-1.04 (1.72)	-.60 (1.36)	.88 (1.18)	.76 (1.19)
	RT	60689.67 (30683.93)	52653.86 (17910.98)	29656.21 (4999.95)	29248.65 (3207.89)
	Accuracy	5.54 (2.13)	5.77 (2.18)	7.17 (1.96)	6.85 (1.97)
**Nutrition**	BIS	-.88 (1.52)	-.35 (1.47)	.50 (1.26)	.73 (1.25)
	RT	46139.76 (19074.39)	40038.20 (15679.32)	25587.92 (922.04)	25565.74 (797.20)
	Accuracy	4.56 (1.25)	4.79 (1.25)	5.04 (1.05)	5.13 (.91)
**Reading**	RT	940.73 (376.52)	770.66 (217.89)	440.94 (16.29)	439.56 (17.48)
**Search**	RT	17418.54 (9868.55)	16154.17 (6568.69)	5707.13 (4184.43)	4852.84 (3248.14)

Table S2 shows that older adults consistently produced poorer performance, longer response times and lower accuracy than young adults across the decision-making, reading and information-search tasks. Clear decision-making scenarios improved older adults' performance and response time, while young adults showed no significant decision-making benefit. Accuracy was less affected by clarity, with no significant main effects or interactions.

Table S2. Summary of ANOVA effects and post hoc t-tests examining the effects of age and clarity across tasks (Combined, ADR, Nutrition, Reading, and Search) on BIS, transformed response time (RT), and accuracy.

		Age	Clarity	Age x Clarity	Older: Enhanced vs Standard	Young: Enhanced vs Standard
**Combined**	BIS	**40.66*****	**7.42****	**8.44****	**3.98*****	< 1
		.30	.07	.08	.47	.03
		$4.998\text{x}10^{+6}$	18.48	22.72	13.56	.16
	RT	**91.70*****	**9.63****	**7.81****	**4.17*****	< 1
		.50	.09	.08	.51	.03
		$1.423\text{x}10^{+13}$	39.52	21.60	7.72	.24
	Accuracy	**10.85****	< 1	3.75	-	-
		.10	.004	.04	-	-
		16.02	.26	.65	-	-
**ADR**	BIS	**38.46*****	2.62	**8.40****	**3.19****	< 1
		.29	.03	.08	.38	.18
		$1262\text{x}10^{+6}$	2.83	10.02	3.60	.31
	RT	**66.63*****	**7.44****	**6.07***	**3.67****	< 1
		.42	.07	.06	.38	.12
		$8.520\text{x}10^{+9}$	8.90	8.79	3.38	.21
	Accuracy	**11.89*****	< 1	3.20	-	-
		.11	.001	.03	-	-
		24.79	.19	.46	-	-
**Nutrition**	BIS	**21.55*****	**11.72*****	1.82	**-**	-
		.19	.11	.02	-	-
		1391.86	26.03	1.98	-	-
	RT	**70.92*****	**4.59***	**4.53***	**3.02****	< 1
		.43	.05	.05	.31	.03
		$1.903\text{x}10^{+10}$	2.05	3.31	1.25	.16
	Accuracy	**3.99***	2.12	< 1	-	-
		.04	.02	.01	-	-
		2.84	.31	.18	-	-
**Reading**	RT	**117.32*****	**14.53*****	**14.07*****	**4.44*****	1.25
		.56	.14	.13	.55	.02
		∞	1875.85	294.70	53.63	.49
**Search**	RT	**89.47*****	**4.69***	< 1	**-**	-
		.49	.05	.002	-	-
		$1.188\text{x}10^{+12}$	1.07	.50	-	-

Note. For each ANOVA effect, the F-value and significance level (*p* < .05*, **p < .01, ***p < .001), partial eta squared (η_p^2), and Bayes Factor (BF_{10}) are reported (from top to bottom). For post hoc comparisons (Enhanced vs. Standard clarity within each age group), the *t*-value and Cohen's *d* are reported. Infinite Bayes Factors are denoted by ∞.

What predicts decision-making ability and the effect of clarity?

Multiple linear regressions were conducted to identify whether education, socioeconomic status (SES), numeracy, vocabulary, and processing speed predicted decision-making performance (average BIS scores across standard and enhanced conditions) and the amount of benefit participants received from improved perceptual clarity (difference between BIS scores in the enhanced and standard conditions). The manuscript reports regression results across all participants. Here we present results separately for the older and young age groups.

Older

The overall model predicting average BIS was significant, $F(5, 41) = 6.47$, $p < .001$, and explained 47.3% of the variance in BIS scores ($R^2 = .473$, adjusted $R^2 = .400$). Better average BIS scores in the older age group were associated with higher numeracy scores ($B = 2.801$, $\beta = .418$, $p = .003$), higher vocabulary scores ($B = 3.757$, $\beta = .304$, $p = .038$), and faster processing speed ($B = 0.169$, $\beta = .382$, $p = .004$). BIS scores were not significantly predicted by educational attainment ($B = 0.055$, $\beta = .086$, $p = .549$) or socioeconomic status ($B = 0.022$, $\beta = .035$, $p = .804$) in this age group. There were no significant predictors of the amount of improvement in BIS score with clearer perceptual information, and the overall model was not significant ($F(5, 41) = 1.69$, $p = .162$).

Young

The same multiple regressions were conducted as above. The overall model predicting average BIS was significant, $F(5, 32) = 4.42$, $p = .005$, and explained 45% of the variance in BIS scores ($R^2 = .450$, adjusted $R^2 = .348$). Better average BIS scores in the young age group were significantly associated with higher numeracy scores ($B = 3.395$, $\beta = .620$, $p = .002$). BIS scores were not significantly predicted by vocabulary scores ($B = 1.201$, $\beta = .132$, $p = .386$), processing speed ($B = 0.024$, $\beta = .064$, $p = .733$), educational attainment ($B = -0.073$, $\beta = -.100$, $p = .515$), or socioeconomic status ($B = 0.045$, $\beta = .097$, $p = .515$) in this age group. There were no significant predictors of the amount of improvement in BIS score

with clearer perceptual information, and the overall model was not significant ($F(5, 32) = 1.47$, $p = .233$).

Research Materials

See Open Science Framework for all decision-making questions (https://osf.io/myb3g). In addition, all tasks were implemented and run using the Gorilla Experiment Builder (https://gorilla.sc/) and include JavaScript code. Access to all task materials is available upon reasonable request. For enquiries, please contact Christopher Atkin (christopher.atkin02@ntu.ac.uk).

References

Liesefeld, H. R., & Janczyk, M. (2019). Combining speed and accuracy to control for speed-accuracy trade-offs (?). *Behavior Research Methods*, *51*(1), 40-60. https://doi.org/10.3758/s13428-018-1076-x

Love, J., Selker, R., Marsman, M., Jamil, T., Dropmann, D., Verhagen, A., & Wagenmakers, E. J. (2015). JASP (Version 0.7) [computer software]. JASP Project.

Madden, D. J., Pierce, T. W., & Allen, P. A. (1992). Adult age differences in attentional allocation during memory search. *Psychology and Aging*, 7(4), 594. https://doi.org/10.1037//0882-7974.7.4.594

Wetzels, R., Matzke, D., Lee, M. D., Rouder, J. N., Iverson, G. J., & Wagenmakers, E. J. (2011). Statistical evidence in experimental psychology: An empirical comparison using 855 t tests. *Perspectives on Psychological Science*, *6*(3), 291-298. https://doi.org/10.1177/1745691611406923
